# Diagnostic Accuracy of Preoperative Neutrophil-to-Lymphocyte and Platelet-to-Lymphocyte Ratios in Detecting Occult Papillary Thyroid Microcarcinomas in Benign Multinodular Goitres

**DOI:** 10.1155/2018/3470429

**Published:** 2018-04-23

**Authors:** Dimitrios K. Manatakis, Sophia Tseleni-Balafouta, Lazaros Tzelves, Dimitrios Balalis, Adelais Tzortzopoulou, Dimitrios P. Korkolis, George H. Sakorafas, Emmanouil Gontikakis, Georgios Plataniotis

**Affiliations:** ^1^First Department of Surgical Oncology, St. Savvas Cancer Hospital, Athens, Greece; ^2^Department of Pathology, School of Medicine, National and Kapodistrian University of Athens, Athens, Greece

## Abstract

**Objective:**

To investigate the diagnostic accuracy of neutrophil-to-lymphocyte (NLR) and platelet-to-lymphocyte (PLR) ratios in detecting occult papillary thyroid microcarcinomas in benign, multinodular goitres.

**Methods:**

397 total thyroidectomy patients were identified from the institutional thyroid surgery database between 2007 and 2016 (94 males, 303 females, mean age 53 ± 14.5 years). NLR and PLR were calculated as the absolute neutrophil and absolute platelet counts divided by the absolute lymphocyte count, respectively, based on the preoperative complete blood cell count.

**Results:**

NLR was significantly higher in carcinomas and microcarcinomas compared to benign pathology (*p* = 0.026), whereas a direct association could not be established for PLR. Both NLR and PLR scored low in all parameters of diagnostic accuracy, with overall accuracy ranging between 45 and 50%.

**Conclusions:**

As surrogate indices of the systemic inflammatory response, NLR and PLR are inexpensive and universally available from routine blood tests. Although we found higher NLR values in cases of malignancy, NLR and PLR cannot effectively predict the presence of occult papillary microcarcinomas in otherwise benign, multinodular goitres.

## 1. Introduction

While ultrasonographic characteristics and fine-needle aspiration (FNA) are the sine qua non of thyroid nodule investigation, papillary thyroid microcarcinomas (PTMC, tumours ≤1 cm along their largest diameter by the WHO definition) not infrequently escape preoperative diagnosis and are only revealed in the final histology report [1]. These occult papillary microcarcinomas constitute the majority of incidentally discovered thyroid malignancies in patients undergoing surgery for presumably benign disease, with a reported incidence of up to 15% [1]. Although they generally carry excellent prognosis, a small percentage represents aggressive tumours with unfavourable patient outcomes [2, 3].

Based on the hypothesis that tumours cause a systemic inflammatory response, which can be expressed by the neutrophil-to-lymphocyte ratio (NLR), Seretis et al. were one of the first groups to study the potential association of papillary thyroid carcinoma and NLR and proposed that NLR may predict the presence of occult PTMC in otherwise benign goitres [4]. Expanding this concept, several researchers studied inflammatory haematological parameters in thyroid cancer, albeit with heterogeneity in statistical methodology and inconsistent results [5–17].

Supported by our previous finding that higher NLR is associated with more aggressive histopathological features in thyroid papillary carcinomas, we aimed to investigate the diagnostic accuracy of neutrophil-to-lymphocyte and platelet-to-lymphocyte ratios in predicting occult PTMC in clinically asymptomatic, FNA-negative thyroid goitres [16].

## 2. Materials and Methods

Patients who underwent thyroidectomy between January 2007 and December 2016 at the First Department of Surgical Oncology, St. Savvas Cancer Hospital, were retrospectively identified from the institutional thyroid surgery database. Demographic characteristics (sex and age), laboratory test results (TSH, total white blood cells, neutrophil and lymphocyte differential counts, and platelets), and pathology outcomes were documented for each patient.

Neutrophil-to-lymphocyte ratio was calculated as the absolute neutrophil count divided by the absolute lymphocyte count, whereas platelet-to-lymphocyte ratio (PLR) was calculated as the absolute platelet count divided by the absolute lymphocyte count, based on the preoperative complete blood cell count. According to our preoperative assessment protocol, fasting baseline blood samples are routinely obtained between 08:00 and 10:00 am on the day before surgery and include haematocrit, haemoglobin, total WBC, and automated differential counts (neutrophils, lymphocytes, monocytes, basophils, and eosinophils) and platelets (UniCel DxH 800 cellular analysis system, Beckman Coulter, USA). This standardised protocol contributed to adjusting for the known impact of circulating hormones (circadian rhythm) on the number and distribution of WBC subtypes and platelets [16].

Pathology reports were reviewed independently by two authors to determine final diagnosis, thyroid specimen weight, and maximum tumour diameter (for malignant lesions). Hashimoto's thyroiditis was identified by extensive infiltration of T lymphocytes and plasma cells, as well as formation of germinal centres.

Included in the final statistical analysis were all consecutive patients, aged ≥18 years, who underwent total thyroidectomy either for benign multinodular goitre or for papillary carcinoma. Patients with follicular, poorly differentiated, medullary, and anaplastic carcinomas as well as hyperthyroidism of any aetiology were excluded. Also excluded were patients with known haematological disorders, chronic medical conditions affecting WBC and platelet counts, past history of malignancy, acute myocardial infarction, or coronary revascularisation within 6 months before surgery and glucocorticoid administration within 3 months before surgery. Noneuthyroid patients and patients with acute infections or with baseline total WBC or platelets outside the institutional reference range (4,000–10,000/ml and 150,000–450,000/ml, resp.) were also excluded.

On the basis of histological diagnosis, eligible patients were categorised into 4 subgroups (multinodular goitre, group MNG; Hashimoto's lymphocytic thyroiditis, group HLT; papillary thyroid microcarcinoma, group PTMC; papillary thyroid carcinoma, group PTC), which were then compared in terms of clinical, pathological, and biochemical parameters.

In order to assess the role of NLR and PLR as possible biomarkers for occult PTMC in otherwise benign multinodular goitres, subgroups MNG and PTMC were unified and then stratified by the NLR and PLR values. We excluded patients with Hashimoto's thyroiditis, to avoid a potential confounding factor. Using receiver operating characteristic (ROC) curve analysis, we calculated sensitivity, specificity, positive and negative predictive values, and accuracy for various NLR and PLR values (80% and 90% specificity; 80% and 90% sensitivity; specificity = sensitivity; mean and median NLR/PLR), and we tried to establish optimal cut-off values for the diagnosis of occult microcarcinoma.

Continuous variables were expressed as mean ± standard deviation, while categorical variables were expressed as percentages. Statistical analysis was performed on MedCalc for Windows, version 17.9.7 (MedCalc Software, Ostend, Belgium), using Student's *t*-test, ANOVA, and chi square criteria. Statistical significance was set to *p* < 0.05. The study was designed following the Standards for Reporting of Diagnostic Accuracy Studies (STARD guidelines, http://www.equator-network.org/reporting-guidelines/stard) and was approved by the ethics committee.

## 3. Results

During the course of the study period, 423 patients underwent total thyroidectomy in our department. Of those, 397 patients fulfilled the inclusion criteria and were included in the final analysis. Demographic characteristics and haematological data are shown in Table 1. Comparative data of the 4 subgroups are depicted in Table 2.

The subgroups did not differ in terms of gender distribution; however, PTC patients were younger than the other groups (*p* = 0.04). Thyroidectomy specimen weight was also significantly different (*p* = 0.01), however without an association between benign and malignant histology (HLT < PTMC < PTC < MNG). The groups were comparable in terms of TSH values, total WBC, and lymphocyte and platelet counts. Neutrophil counts were significantly different, with higher counts in groups PTMC and PTC (*p* = 0.015).

**Figure 1:**
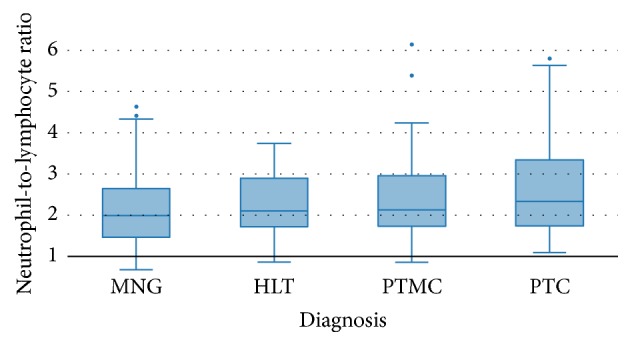
Comparison of neutrophil-to-lymphocyte ratio (mean ± SD) among the subgroups MNG, HLT, PTMC, and PTC.

**Figure 2:**
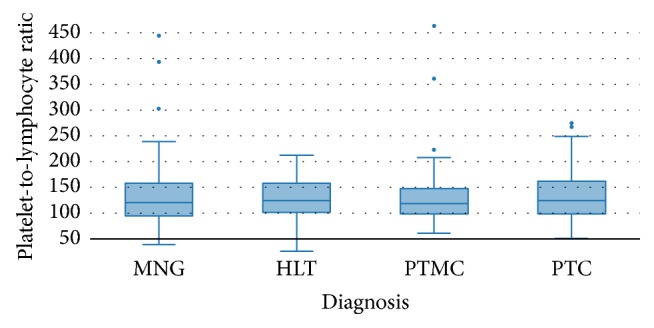
Comparison of platelet-to-lymphocyte ratio (mean ± SD) among the subgroups MNG, HLT, PTMC, and PTC.

Neutrophil-to-lymphocyte ratio was significantly different among the 4 groups, with progressively higher values in benign goitres, Hashimoto thyroiditis, microcarcinomas, and carcinomas (*p* = 0.026) (Figure 1). On the other hand, PLR values also differed significantly among the 4 groups, although a clear association could not be established (HLT < PTMC < MNG < PTC, *p* = 0.0002) (Figure 2).

**Figure 3:**
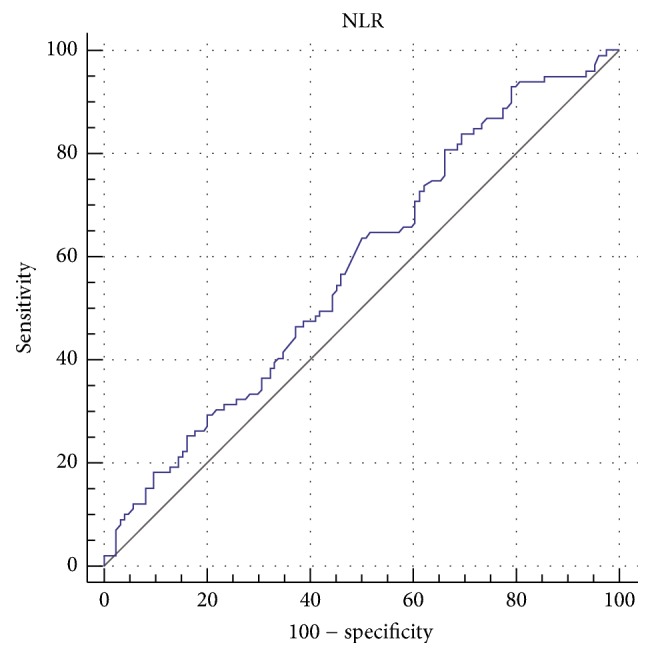
ROC curve for neutrophil-to-lymphocyte ratio.

**Figure 4:**
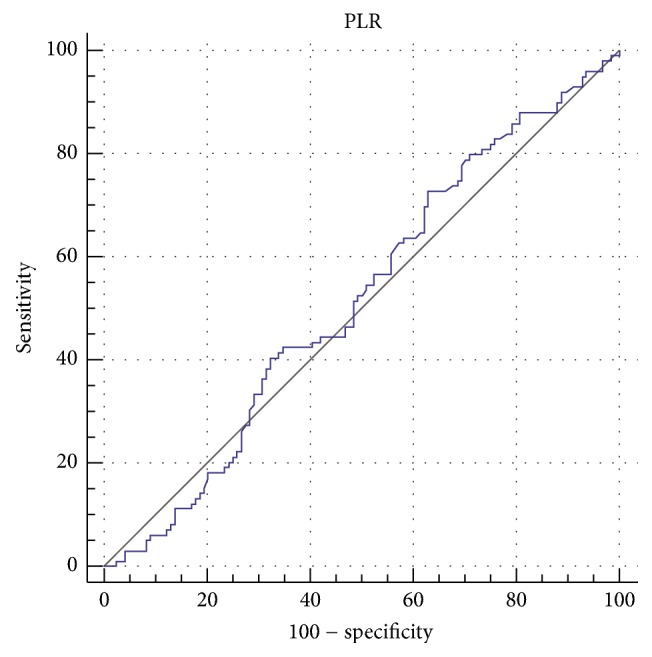
ROC curve for platelet-to-lymphocyte ratio.

In the ROC curve analysis, both NLR and PLR fared poorly. For the NLR, the area under the ROC curve (AUC) was 0.578 (95% confidence interval 0.510 to 0.644) (Figure 3). The optimal cut-off value was calculated at 1.67, achieving sensitivity of 80% and specificity of 33.9% (Table 3). On the other hand, AUC for PLR was 0.518 (95% confidence interval 0.450 to 0.585) (Figure 4) and the optimal cut-off was calculated at 139, achieving sensitivity of 73% and specificity of 37% (Table 4). Both NLR and PLR scored low in all parameters, with overall accuracy ranging between 45 and 50%.

## 4. Discussion

The prevalence of thyroid cancer worldwide is increasing steadily over the past three decades [1, 3, 18]. It is estimated that each year 65,000 new cases will be diagnosed in the USA, with around 2,000 cancer-related deaths. The increased detection rate has been partly attributed to improvements in imaging techniques, which have led to detection of smaller, subclinical tumours [3, 18, 19]. This PTMC “epidemic” presents not only a management dilemma, but also a public health issue [3].

Incidentally discovered papillary microcarcinomas have been observed in 2–15% of benign, multinodular goitres and in up to 35% of cases in autopsy series [20]. They are usually characterised by slow growth, minimal invasiveness, and indolent course, with low metastatic potential and excellent prognosis [19, 21, 22]. Whether incidentally and nonincidentally diagnosed PTMC are essentially different entities, requiring different management, is still a subject of debate [23]. The notion that the two subgroups are distinct entities may be supported by the observation that size, gender distribution, and recurrence probability are different between the two groups [19]. On the other hand, long-term prognosis remains excellent for both [19, 23].

Sugitani et al. have proposed a classification of PTMC into 3 biologically different types, which require different approaches. Type I (90–95%) represents the lowest-risk, asymptomatic PTMC, for which active surveillance is feasible. Type II (5–10%) comprises an earlier stage of the usual, low-risk PTC and can be treated by conservative surgery. Type III (<1%) includes high-risk, clinically symptomatic PTMC, with extrathyroidal invasion and lymph node metastases, requiring aggressive surgery [2, 3].

Whereas ultrasonography and FNA biopsy are the mainstays of thyroid nodule diagnostic work-up, up to 30% of biopsies may be misleading and result in unnecessary surgical explorations [24]. Noninvasive genomic and proteomic biomarkers for microcarcinomas are currently under development, to improve FNA accuracy and reduce false-negative results [24]. BRAF V600E mutational status, galectin-3, E-cadherin, CD44v6, angiogenic (HIF-1*α*, VEGF), and antiangiogenic factors (PEDF) have been studied and results are certainly promising [24]. However they are still complex in their methodology and interpretation and certainly not universally available.

The role of simple haematological parameters, like neutrophil-to-lymphocyte ratio, platelet-to-lymphocyte ratio, and mean platelet volume (MPV) was also investigated [4–16]. The rationale behind is that these indices are a quantitative expression of the systemic inflammatory response and reflect the link between tumorigenesis and chronic inflammation [25]. High NLR values occur due to either neutrophilia or lymphopenia. Neutrophilia affects the immune system by suppressing the cytolytic capability of lymphocytes, activated T cells, and natural killer cells [26]. On the other hand, the importance of lymphocytes is highlighted by the fact that tumours with dense lymphocytic infiltration tend to have better overall prognosis [26]. As for platelets, they also have a key role in the process of malignant transformation and metastatic spread. Indeed, thrombocytosis has been related to poor prognosis in many types of cancer and larger platelets are more metabolically and enzymatically active, as reflected on the MPV [12, 15]. Common denominator in all three conditions, neutrophilia, lymphopenia, and thrombocytosis, is a dysregulation in the secretion of cytokines, chemokines, and growth factors, produced both by tumour cells and by associated host cells of the tumour microenvironment (cancer-associated fibroblasts, tumour-associated macrophages, mast cells, dendritic cells, myeloid-derived suppressor cells, and B cells) [27].

A pilot study by Seretis et al. was the first study to suggest that NLR could in fact diagnose the presence of thyroid microcarcinomas in benign goitres. In a small series of 109 patients, they found a clear elevation of NLR in papillary and micropapillary carcinomas, compared to benign goitres and controls, although no cut-off was proposed [4]. Multiple subsequent studies however obtained controversial results. Kocer et al. and Kim et al. demonstrated that patients with malignant tumours had increased NLR values in comparison with multinodular goitre and Hashimoto thyroiditis, whereas Liu et al. showed this difference for patients over 45 years of age [6, 8, 11]. Baldane et al. found that MPV was significantly elevated in cases of PTC compared to benign goitres and healthy controls and additionally that MPV values returned to normal postoperatively [12].

On the other hand, several researchers did not detect any trends in NLR, PLR, or MPV between PTC and MNG patients [5, 7, 9, 14, 15]. Moreover, the only meta-analysis of NLR in differentiated thyroid cancer has also failed to show any significant difference between benign and malignant disease. The authors concluded that* “the NLR of patients with differentiated thyroid cancer is not significantly different from that of patients with benign nodules. An elevated NLR seems not a reliable indicator of progressing differentiated thyroid cancer in patients with goitres”* [17].

So far, only Seretis et al. have looked into the role of these inflammatory biomarkers as predictors of PTMC [4]. Our results however do not confirm their findings. Although we did find significantly increased NLR values in PTMC and PTC patients, this was not established for PLR as well. Moreover, the ROC curve analysis revealed that both variables achieved low AUC scores (tests with AUC ≤ 0.75 are generally considered not clinically useful). To overcome this, two cut-off values may be suggested, one with high sensitivity and one with high specificity. Since detection of occult microcarcinomas is the goal, high sensitivity is required, at the cost of specificity.

An interpretation of our outcomes could be that chronic inflammation may be less important in thyroid carcinogenesis, as well as the indolent nature of papillary microcarcinoma, which causes a less vigorous systemic inflammatory response [5, 16]. A critical approach to these inflammatory indices would come to the conclusion that they are largely nonspecific and require careful selection of patients with strict exclusion criteria, since they are affected by a plethora of medical conditions, diseases, and medications (acute and chronic infections, cardiovascular events, allergic reactions, malignancy, corticosteroid and nonsteroid anti-inflammatory drug administration, etc.) [16]. Moreover, constitutional differences among individuals, for example, different HLA subtypes, may lead to variable systemic inflammatory responses to various exogenous and endogenous stimuli. Nevertheless, the main advantage remains that these indices are universally available and easy to extract from routine preoperative blood tests and thus do not increase hospital costs [4, 16].

Limitations of the study protocol include its single-institution, retrospective nature, and average sample size. Furthermore, we could not address the issue of NLR/PLR in conjunction with patient prognosis [16]. Given the excellent overall and disease-free survival of PTMC patients, a long-term follow-up is required to draw safe, meaningful conclusions. Implications for future study may include the possible correlation of systemic inflammatory indices and tumour immune cell infiltration, as well as postthyroidectomy alterations in these parameters.

## 5. Conclusion

Malignant tumours cause a systemic inflammatory response that can be expressed and quantified by indices, such as neutrophil-to-lymphocyte and platelet-to-lymphocyte ratios. Whereas NLR was found significantly increased in patients with papillary carcinomas and microcarcinomas, compared to benign pathology, a clear association could not be established for the PLR as well. Moreover, both biomarkers performed poorly in the diagnostic accuracy analysis and could not effectively predict the presence of occult papillary microcarcinomas in otherwise benign, multinodular goitres.

## Figures and Tables

**Table 1:** Demographic and haematological data.

*N*	397	
Sex		
Male	94	23.7%
Female	303	76.3%
Age (years)	53.01 ± 14.5	Range 18–89
Thyroid specimen weight (gr)	34 ± 27	Range 5–329
TSH (mIU/L)	1.24 ± 1.09	Range 0.29–4.19
WBC (cells/ml)	7265 ± 2025	Range 4200–10000
Neutro (cells/ml)	4485 ± 1650	Range 1800–9100
Lympho (cells/ml)	2066 ± 630	Range 900–5200
Platelets (cells ×1000/ml)	251 ± 67	Range 151–444
NLR (mean ± SD)	2.32 ± 0.95	Range 0.68–6.14
PLR (mean ± SD)	132 ± 54	Range 39–463

**Table 2:** Comparison of subgroups MNG, HLT, PTMC, and PTC.

	MNG	HLT	PTMC	PTC	*p* value
*N*	160	30	113	94	
Sex					
Male	35 (21.8%)	2 (6.7%)	31 (27.4%)	26 (27.7%)	
Female	125 (78.1%)	28 (93.3%)	82 (72.6%)	68 (72.3%)	0.08
Age (years)	55.4 ± 14.2	52.4 ± 12.4	52.2 ± 13.6	50.2 ± 16.1	**0.04**
Thyroid weight (gr)	38.9 ± 31.4	22 ± 12	28.9 ± 21.5	37.2 ± 49.9	**0.01**
TSH (mIU/L)	1.09 ± 0.76	1.44 ± 0.96	1.45 ± 1.51	1.40 ± 1.45	0.1
WBC (cells/ml)	6916 ± 1845	7230 ± 1813	7421 ± 2161	7691 ± 2146	0.06
Neutro (cells/ml)	4138 ± 1476	4423 ± 1477	4686 ± 1850	4854 ± 1614	**0.015**
Lympho (cells/ml)	2056 ± 586	2069 ± 663	2051 ± 558	2109 ± 789	0.94
Platelets (cells ×1000/ml)	249 ± 71	254 ± 79	246 ± 66	256 ± 59	0.83
NLR (mean ± SD)	2.14 ± 0.85	2.26 ± 0.79	2.40 ± 0.96	2.54 ± 1.10	**0.026**
PLR (mean ± SD)	131 ± 58	128 ± 44	129 ± 55	134 ± 50	**0.0002**

**Table 3:** Diagnostic accuracy analysis for neutrophil-to-lymphocyte ratio.

NLR	Sens	95% CI	Spec	95% CI	PPV	NPV	Accuracy
1.39	90%	82.2–95.0	21%	14.2–29.2	47.6	72.2	43.8%
1.67	80%	71.7–88.0	34%	25.6–42.9	49.4	68.9	45.1%
2.05	55%	44.2–64.6	54%	44.9–63.0	48.6	59.8	44.7%
2.06	55%	44.2–64.6	55%	45.7–63.8	49.1	60.2	44.7%
2.40	40%	29.7–49.7	67%	57.9–75.1	48.7	58.0	44.7%
2.83	27%	18.8–37.1	80%	71.7–86.5	51.9	57.9	45.0%
3.27	18%	11.1–27.2	90%	83.7–94.9	60.0	58.0	44.9%

**Table 4:** Diagnostic accuracy analysis for platelet-to-lymphocyte ratio.

PLR	Sens	95% CI	Spec	95% CI	PPV	NPV	Accuracy
77	6%	2.3–12.7	90%	84.7–95.5	35.3	54.9	46.6%
85	17%	10.3–26.1	80%	71.7–86.5	40.5	54.7	46.6%
119.5	51.5%	41.3–61.7	51.5%	42.5–60.7	45.9	57.1	47.0%
120	51.5%	41.3–61.7	50%	41.7–59.9	45.5	56.8	48.9%
130	62%	51.3–71.2	43.5%	34.7–52.7	46.6	58.7	48.9%
139	73%	62.9–81.2	37%	28.6–46.2	48.0	63.0	48.9%
156	80%	70.5–87.2	26%	19.1–35.3	46.5	62.3	47.1%
186	90%	82.2–95.0	11%	6.3–18.2	44.7	58.3	47.1%
